# Temperate Coastal Microbial Communities Rapidly Respond to Low Concentrations of Partially Weathered Diesel

**DOI:** 10.1007/s00248-021-01939-w

**Published:** 2021-12-10

**Authors:** Camilla M. Ryther, Alice C. Ortmann, Gary Wohlgeschaffen, Brian J. Robinson

**Affiliations:** 1 Biology Department, Dalhousie University, 6299 South Street, Halifax, NS B3H 4R2 Canada; 2 Centre for Offshore Oil, Gas and Energy Research Laboratory, Bedford Institute of Oceanography, 1 Challenger Drive, Dartmouth, NS B2Y 4A2 Canada

**Keywords:** Diesel, Petroleum weathering, Microbial community diversity, Phytoplankton, Mesocosm, Bacteria

## Abstract

Diesel is frequently encountered in coastal ecosystems due to land run-off from road surfaces. The current study investigates how partially weathered diesel at environmentally relevant concentrations, as may be seen during a run-off event, affect coastal microbial communities. A mesocosm experiment using seawater from the Bedford Basin, Nova Scotia, was followed for 72 h after the addition of partially weathered diesel. Sequencing data suggests partially weathered diesel acts quickly to alter the prokaryotic community, as both opportunistic (*Vibrio* and *Lentibacter*) and oil-degrading (*Colwellia*, *Sulfitobacter*, and *Pseudoalteromonas*) bacteria proliferated after 24 h in comparison to the control. In addition, total prokaryotes seemed to recover in abundance after 24 h, where eukaryotes only ceased to decrease slightly at 72 h, likely because of an inability to adapt to the oil-laden conditions, unlike the prokaryotes. Considering there were no highly volatile components (benzene, toluene, ethylbenzene, and xylene) present in the diesel when the communities were exposed, the results indicate that even a relatively small concentration of diesel run-off can cause a drastic change to the microbial community under low energy conditions. Higher energy conditions due to wave action may mitigate the response of the microbial communities by dilution and additional weathering of the diesel.

**Supplementary Information:**

The online version contains supplementary material available at 10.1007/s00248-021-01939-w.

## Introduction

Diesel is commonly used in transportation, industrial, and recreational activities and can enter the marine ecosystem from run-off [1]. Oil introductions are especially concerning in coastal ecosystems, which contribute most to net primary production in comparison to the rest of the ocean, due to phytoplankton, including picoeukaryotes and prokaryotic plankton [2]. Diesel is a light distillate oil mostly composed of hydrocarbons between C_8_ and C_24_, including alkanes, monocyclic aromatic hydrocarbons (MAHs), and polycyclic aromatic hydrocarbons (PAHs). Alkanes are a known substrate for oil-degrading bacteria [3], making most diesel accessible for degradation and consumption [1, 4]. MAHs include benzene, toluene, ethylbenzene, and xylene (BTEX), which have acute toxic effects on many organisms, including plankton, due to their water solubility and high volatility [5]. Specific PAHs found in diesel, like naphthalene, have been linked to decreases in primary production [2]. Light distillates can enter the water column readily, even under low energy conditions, increasing exposure of the microbial community [1]. Thus, specific compounds in the oil and the extent of weathering are crucial for understanding the ramifications of potential exposure to microbes.

Once petroleum products enter the environment, weathering processes occur quickly, causing chemical and physical changes. Experiments measuring microbial responses have focused on the impacts of fresh oil [6–10], which simulates real-time accidental releases into seawater. Another approach uses artificially weathered oil [11], where oil is manipulated mechanically for a set amount of time under controlled temperature and light conditions [2], resulting in dissolved or dispersed petroleum hydrocarbons in the water accommodated fraction [12]. Since weathering causes direct changes in oil composition, like loss of small alkanes and volatiles, utilization of fresh oil is unlikely to provide insight into microbial responses to oil that has already been altered. Artificial weathering attempts to overcome this; however, excessive losses of volatile compounds can occur, resulting in extensively weathered products. These highly weathered products may not reflect short weathering times that may be associated with run-off from road surfaces into coastal environments.

Considering primary producers and plankton are the base of the food web, understanding their response to oil introductions can help predict impacts at higher trophic levels. Following introductions of oil, bacterial communities tend to shift, with certain species dominating in oil-laden conditions which can contribute to hydrocarbon degradation. Community shifts have been observed across a variety of oil types, from heavier crudes to lighter distillates [13–15]. Chronopoulou et al. [14] found that the dominant bacteria, SAR11 (Pelagibacteracea), was replaced by members of the genus *Pseudoalteromonas*, which dominated plume samples following the *Deepwater Horizon* oil spill [16]. Many bacteria, such as *Pseudomonas* and *Colwellia*, have been found to degrade BTEX in marine environments and noted as oil-degrading specialists [17, 18]. Additional bacterial taxa known to degrade oil include the aliphatic degraders, *Alcanivorax* and *Marinobacter* as well as the PAH-degrading *Alteromonas* and *Cycloclasticus* [14]. Oil-degrading bacterial communities, as well as native communities, vary in diversity and community structure depending on location and season [13, 19–21].

This study aims to characterize the effects of partially weathered diesel at environmentally relevant concentrations on a coastal microbial community. Complimenting previous studies measuring the response of microbial communities to fresh [7, 13] and artificially weathered diesel [12], partially weathered diesel, including some, but not all, volatile hydrocarbons, was utilized. Specifically, we used a more natural approach to weather diesel for 24 h and then monitored the abundance and diversity of coastal microbial communities exposed to the oil over time compared to unoiled controls. The results address a gap in the literature about entire communities and what may happen when partially weathered diesel enters a coastal ecosystem, which may aid in identifying appropriate mitigation techniques.

## Methods

### Experimental Setup

A small-scale mesocosm experiment was conducted at the Bedford Institute of Oceanography (BIO) in Dartmouth, Nova Scotia, in November 2019. The experiment was set up outdoors, protected from precipitation, but with exposure to natural light, temperature, and wind. Three flow-through incubators contained three enclosures each, with three treatments: control (no oil added), high concentration (0.18 mL L^−1^), and low concentration of weathered diesel (0.07 mL L^−1^), representing a split-plot design. Concentrations were selected to be similar to previous experiments using fresh diesel [7]. Enclosures were 5-gallon plastic buckets lined with polytetrafluoroethylene plastic bags filled with 14 L of unfiltered water taken directly from the Bedford Basin at a depth of 2.5 m below the surface. HOBO UA-002–08 Pendant Temperature/Light Data Loggers were secured to sampling pipes in each enclosure, where aquarium air pumps delivered air, preventing deoxygenation and stratification. To regulate the temperature, enclosures were placed in flow-through incubators using the municipal water supply.

Dyed marine diesel (MD) was purchased from Bluewave Energy (https://www.bluewaveenergy.ca/). For weathering, 30 mL of MD was added to 3–250 mL glass dishes with a diameter of 10 cm and weighed. Dishes were set on top of the middle enclosure for each incubator. After 24 h of exposure to represent a short weathering window that may occur after a rain event, dishes were re-weighed and samples from each were collected for gas chromatography–mass spectrometry (GCMS), density, and viscosity analyses. Following subsampling, diesel was added to enclosures using a glass syringe.

### Sampling and Analyses

Samples were collected at 0 h, 6 h, 12 h, 24 h, 48 h, and 72 h post-oiling. This short-time frame was chosen to minimize potential bottle effects and to ensure that only ~ 20% of the total volume was removed during sampling. Temperature, salinity, and dissolved oxygen (DO) were measured with a Professional Plus 2030 probe (YSI). Using silicone tubing and a peristaltic pump, BTEX samples were collected in a purge and trap vial containing 40 µL of 1 N HCl and total petroleum hydrocarbon (TPH) samples were collected in a glass amber bottle. Dichloromethane was added to each TPH sample, mixed thoroughly, and refrigerated until quantification. Water for subsampling as described below was collected in an autoclaved glass bottle. At 72 h, water samples were collected for GCMS analysis following the protocols for TPH samples.

BTEX was quantified using an Agilent 6890 GC coupled with a purge and trap concentrator and detected with an Agilent 5973 MS [22]. TPH samples were analyzed using GC-flame ionization detection (GC-FID) protocols outlined in King et al. [23]. Concentrations of *n*-alkanes and PAHs in fresh MD, weathered MD, and water samples collected at 72 h were determined using GCMS analysis. Compounds were isolated using a liquid–liquid extraction before being purified using a solid-phase extraction method and analyzed using GC coupled with MS using Agilent 6890B and Agilent 5975B systems, respectively [22].

Water from the autoclaved bottles was subsampled for biological and inorganic nutrient analysis. Samples for inorganic nutrients were collected in duplicate 10 mL vials and stored at − 20℃. After thawing, samples were measured on an SEAL Analytical AA2 continuous segmented flow autoanalyzer. Values below the detection limit were considered zero for analysis. Nitrate + nitrite, nitrite, ammonium, phosphate, and dissolved silicate were measured directly, and nitrate was determined by subtracting nitrite from the nitrate + nitrite value.

Small phytoplankton (< 5 µm) was preserved in a final concentration of 0.5% EM grade glutaraldehyde and 1% Pluronic ™ F-68 solution [24] for 10 min at 4℃ and stored at − 86℃ until processing [6]. Subsamples for prokaryote and viruses enumeration were preserved in a final concentration of 0.5% glutaraldehyde following the same protocol. Methods in Ortmann et al. [8] were modified and applied to a FACSLyric© flow cytometer (BD Biosciences) equipped with 488 nm and 640 nm lasers. Phytoplankton was analyzed on high flow for 7 min. Prokaryotes and viruses were diluted at 1:10 and 1:100, respectively, in filtered Tris–EDTA (TE, pH = 8), stained with SYBR Green (Lonza), and analyzed on medium flow for 2 (viruses) or 3 (prokaryotes) min for quantification. Fluorescent beads, as internal standards, ensured the instrument was collecting data correctly [6]. FSC files were imported to FlowJo® v 10.6.1 software, and groups of phytoplankton were identified based on fluorescence (Supplementary Fig. S1). The same procedure was followed for prokaryotic and viral FSC files using a green fluorescence *vs*. SSC-H plot.

Microplankton was preserved in a 1% Lugol’s solution. Samples were analyzed using a FlowCam© 8000 with a 4 × objective for 15 min at 2 mL/min. Particles with equivalent spherical diameters between 15 µm and 1500 µm were captured. Files were pre-processed to remove blanks or repeats due to machine errors. Image libraries were created based on a set of 9 random samples for microbial groups that were easily identified, and a training set was created with 8 classes. Classified files were subjected to manual sorting to correct misclassified images, and abundances for each group were calculated as particles/mL.

DNA was extracted from the filters using a QIAGEN MagAttract PowerWater DNA Kit with the KingFisher Duo Prime (Thermo) following the manufacturer’s instructions at the Integrated Microbiome Resource at Dalhousie University, Halifax, NS. The V4-V5 region of the 16S rRNA gene [25] and the V4 region of the 18S rRNA gene [26] were amplified for sequencing using 300 base-pair, paired-end sequencing on an Illumina MiSeq. Demultiplexed FASTQ files were downloaded and processed using the QIIME2 2019.10 pipeline [27]. Briefly, reads were trimmed with cutadapt to remove the primer sequences [28] and denoised using the DADA2 plugin [29] after inspecting read quality to determine trimming parameters. ASVs were assigned taxonomy using a classifier trained to the appropriate rRNA gene region with the SILVA 132 database and a naïve Bayes approach using 99% similarity [30]. The final 16S rRNA gene ASV table was filtered to remove features identified as mitochondria or chloroplasts. The BioProject accession number is PRJNA678694.

### Statistical Analyses

**Fig. 1:**
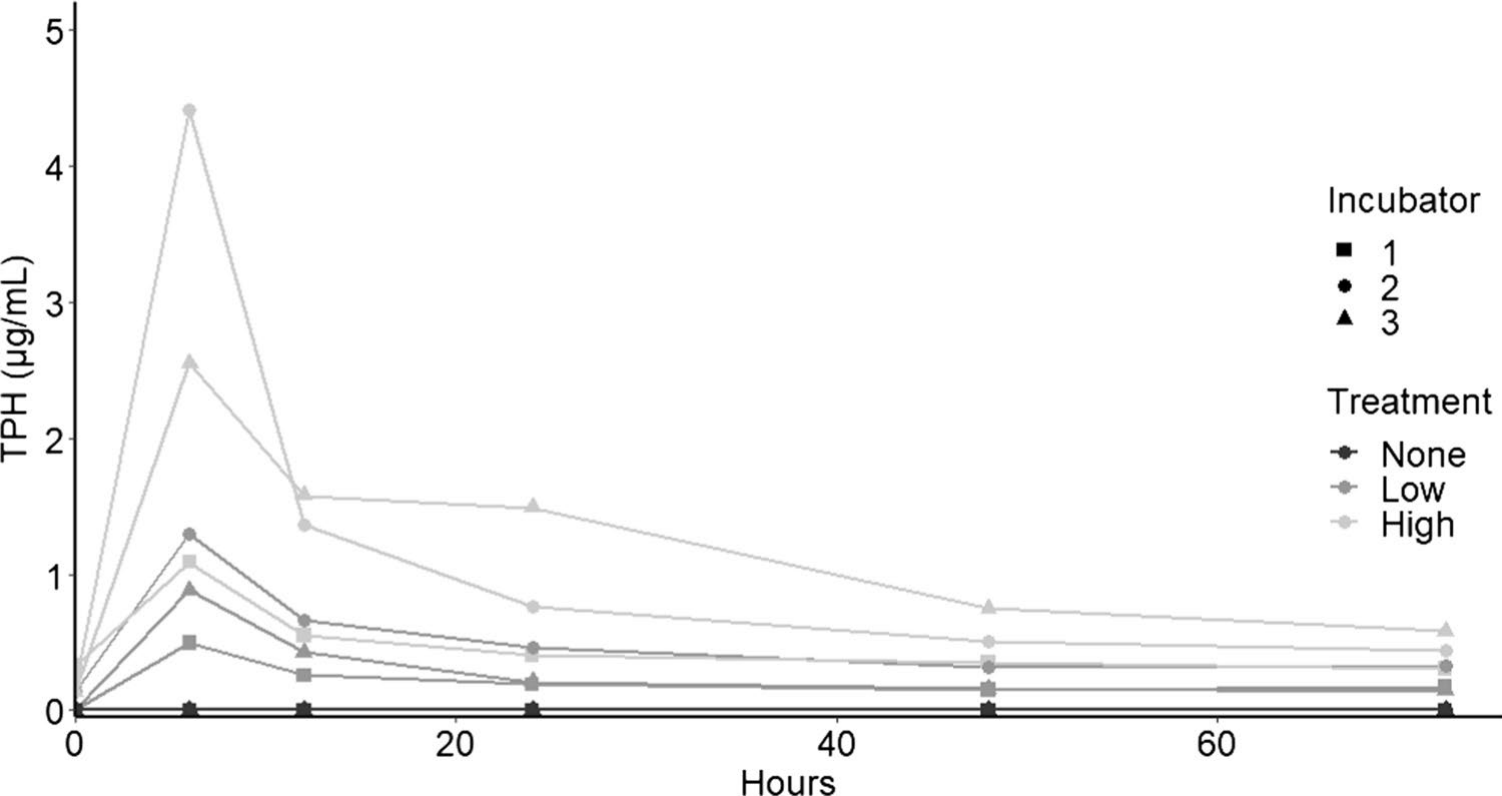
TPH concentrations measured in each enclosure over time

Initial analysis of TPH concentrations indicated that concentrations within the high and low treatments varied among enclosures, especially for the high treatments (Fig. 1). For overall analysis, high and low treatments were combined into an MD treatment reflecting the gradient of TPH concentrations in the experiments. Linear mixed models assessed the effects of time and the addition of partially weathered MD on the abundance of microbial groups and physical and chemical parameters. Analyses were carried out in R (3.6.2; www.r-project.org) and packages ‘lme4’ [31] and ‘lmerTest’ [32]. Enclosures and incubators were included as random variables, with enclosures nested within incubators. A step analysis was done to assess the fit of each model to its respective dependent variable and identify the most appropriate model. For virus and prokaryotic abundances, random effects were excluded due to convergence issues, and a linear model was applied instead. To assess the impact of weathered MD on the individual phytoplankton and microplankton groups, generalized linear models (GLMs) for multivariate data (*GLM*_*mv*_) were fitted according to Szöcs et al. [33]. GLMs were fitted to a negative binomial distribution with time and treatment as predictors. To take the repeated measures design into account, a restricted permutation method using the package ‘permute’ [34] was completed by restricting permutations (set to 1000) within incubators (‘blocks’) and between enclosures (‘plots’). The model without the interaction was compared to the full model using a likelihood-ratio test.

The impact of partially weathered MD on community composition was identified by processing the raw ASV tables using the DEICODE QIIME2 plugin to calculate the Aitchison distance [35]. Default values of 10 and 1000 were used for the minimum feature count and minimum sample count parameters, respectively. The distance matrix was imported into Primer 6 + and analyzed using PERMANOVA [36]. Treatment and time were set as fixed factors, while incubator and enclosure were random factors, with incubator nested in treatment and enclosure nested in the incubator. Interactions between time and enclosure and time and incubator were excluded from the analysis. Figures were plotted using ‘ggplot2’ [37] and ‘ComplexHeatmap’ [38].

## Results

Temperature, DO, and salinity showed no differences among treatments, although all three varied over time. The temperature averaged 11.8℃ ± 0.6 at 0 h and from 24 to 72 h. At 6 h and 12 h, there was a slight increase in temperature with an average of 14.7 ℃ ± 0.3. A decrease in salinity was measured in two enclosures in incubator 3 from 29.9 (± 0.3) to 24.1 (± 1.1) between 0 and 6 h due to an accidental introduction of freshwater from the temperature regulation system. Light intensity was averaged for daylight hours for each enclosure. There were significant daily differences with the highest light on day 3, but no differences between treatments.

Following 24 h of weathering, density and viscosity of the MD increased, where mass loss averaged 22.2% (± 2.24) (Table 1). BTEX was always below the detection limit of 0.5 ng/mL, although fresh diesel has almost 1000 ng mL^−1^ BTEX [39]. Within the treatment enclosures, a range of TPH concentrations was observed, leading to the pooling of high and low concentrations into a single treatment factor. TPH peaked at 6 h in all MD enclosures, decreasing over time **(**Fig. 1).

**Table 1:** The density, viscosity, and % weathering for the fresh MD and the partially weathered MD after 24 h

Sample	Density (g cm^−3)^	Viscosity (cSt)	% Weathering
Fresh MD	0.8098	2.1991	-
Weathered #1	0.8173	2.7406	25.2
Weathered #2	0.8174	2.7425	21.6
Weathered #3	0.8174	2.7592	19.8

% Weathering is the % of mass loss over the 24 h of exposure

**Fig. 2:**
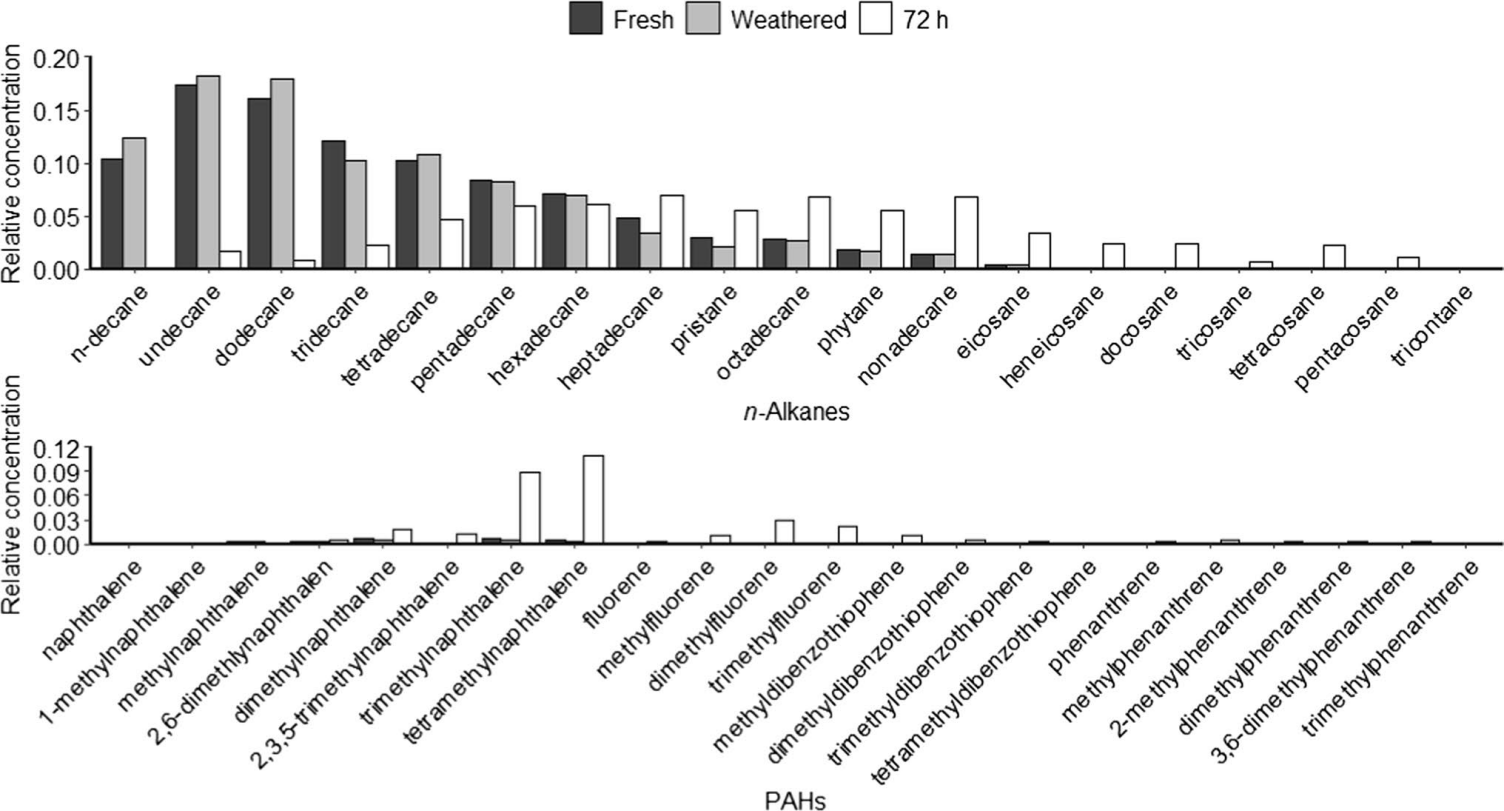
The average relative concentration of *n*-alkanes and PAHs measured by GCMS for fresh MD, MD after 24 h of weathering, and water samples 72 h after addition of weathered MD

GCMS analysis of fresh MD and weathered samples showed differences in concentrations of *n*-alkanes and PAHs (alkylated and parent compounds) between weathered and fresh MD (Fig. 2). Generally, the concentration of measured compounds was higher in the weathered product based on mass, suggesting compounds not measured by GCMS were lost during weathering. Control enclosures had no detectable PAHs at 72 h, although some *n*-alkanes were measured using GCMS (Supplementary Table S1). These *n*-alkanes, C_10_-C_12_, C_17_-C_20_, and C_23_-C_25_, may be biologically produced compounds [40]. In the MD enclosures, there were preferential losses of lighter *n*-alkanes relative to fresh and weathered diesel (Fig. 2), although absolute concentrations varied across enclosures. Trimethylnaphthalene and tetramethylnaphthalene dominated the PAHs at 72 h. Total alkanes relative to total PAHs were lower at 72 h compared to the weathered MD, suggesting degradation of the *n*-alkanes was faster than for the PAHs.

NH_4_^+^, NO_3_^−^, and PO_4_^−3^ all showed a significant interaction between treatment and time (Supplementary Fig. S2). In control enclosures, NO_3_^−^ and PO_4_^−3^ increased slightly over time, while larger increases were observed for NH_4_^+^. The addition of weathered MD had little impact on concentrations until 48–72 h, when concentrations of nutrients decreased. The introduction of freshwater into the two enclosures between 0 and 6 h resulted in immediate decreases in nitrate and ammonium and increases in phosphate concentrations, but the overall patterns after 6 h matched the other enclosures.

**Fig. 3:**
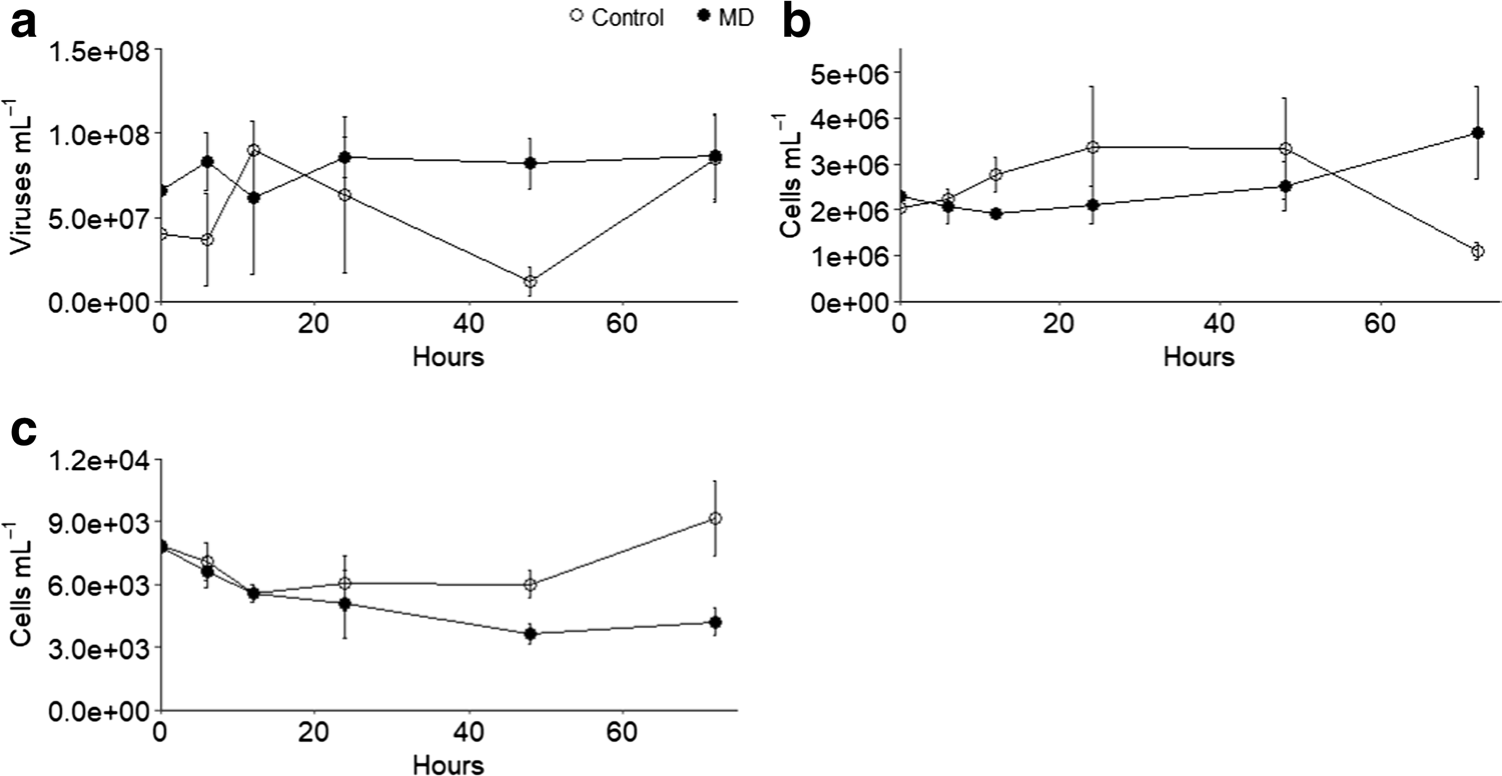
Mean abundances and standard deviations of total viruses (**a**), prokaryotes (**b**), and phytoplankton < 5 μm (**c**) for each treatment

**Fig. 4:**
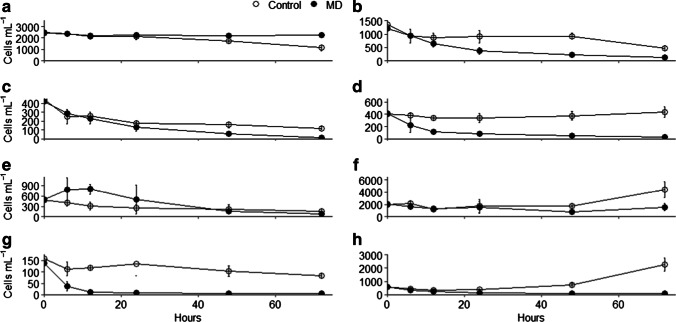
Mean abundances and standard deviations of the 8 groups of small phytoplankton corresponding to the groups identified in Supplementary Fig. S1

Abundances of viruses, prokaryotes, and small phytoplankton (< 5 um) all showed significant interactions between treatment and time, but their overall patterns differed. Virus abundances stayed relatively stable in the presence of weathered MD, while in control enclosures, they decreased by ~ eightfold from a peak at 12 to 48 h before recovering by 72 h (Fig. 3a). Total prokaryotic abundances showed opposite patterns, with immediate increases in control enclosures, but a 12 h lag in increases in the enclosures with MD (Fig. 3b). Total small phytoplankton decreased between 0 and 12 h in both treatments, with patterns diverging after that time (Fig. 3c). Recovery and growth of small phytoplankton was observed in the control enclosures, with no recovery observed in the MD enclosures. The 8 groups likely include multiple taxa, but based on fluorescence, size, amplicon sequencing data, and knowledge of the area, potential taxa are identified. Among the 8 groups, positive, negative, and neutral responses to weathered MD were observed (Fig. 4); however, treatment effects were only significant for groups D (diatoms, possibly *Thalassiosira*) and G (phycocyanin-containing cyanobacteria), where abundances in control enclosures were higher than in the MD for most of the experiment. Although not significant, group E (Cryptomonadales) did show an initial positive response to MD, and groups F (phycocyanin-containing cyanobacteria) and H (Cryptomonadales, Rhodomeniopycidae, or large cyanobacteria) show increasing abundances at 72 h. The most abundant group, A, likely includes phycoerythrin-containing cyanobacteria (*Synechococcus* or *Cyanobium*), whereas B and C are likely different green algae (Prasinophyceae, Chlorophyceae, and Mammiellophyceae).

**Fig. 5:**
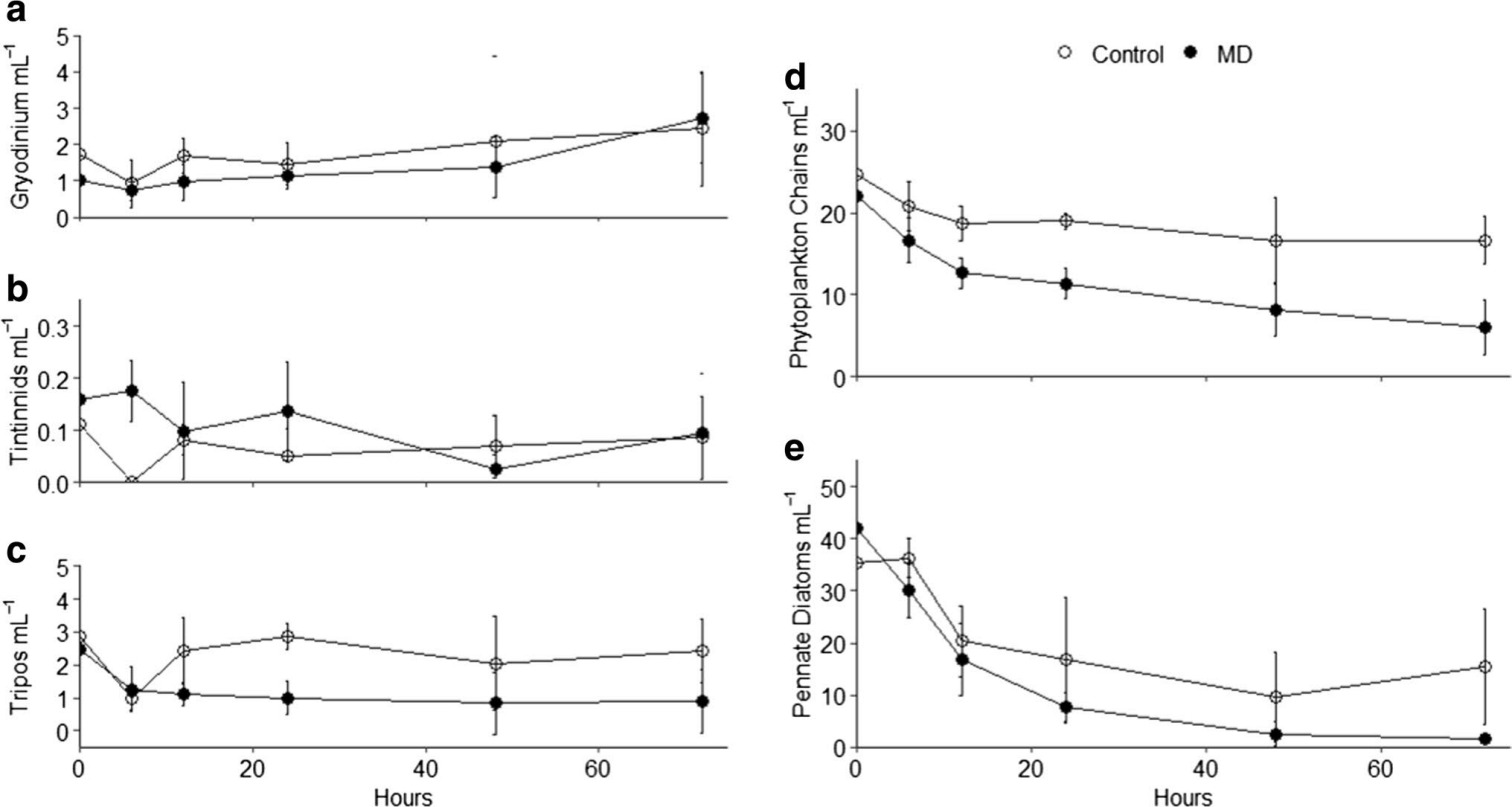
Mean abundance and standard deviations for the 5 groups of microplankton identified in this study

Five groups of microplankton were quantified and included *Tripos*, *Gyrodinium*, tintinnids, phytoplankton chains, and pennate diatoms (Fig. 5). Phytoplankton chains and pennate diatoms were the most abundant across the entire experiment, with abundances of phytoplankton chains significantly higher in control enclosures. Phytoplankton chains included chain-forming cyanobacteria (*Nostoc*, *Nodosilinea*, *Rivularia*, *Leptolyngbya*, and *Trichodesmium*), diatoms (*Chaetoceros*, *Leptocylindrus*, and *Thalassiosira*), and dinoflagellates (*Alexandrium*) based on amplicon sequencing. *Tripos* were also significantly higher in control enclosures compared to weathered MD enclosures, although their abundances were low. Pennate diatoms, likely *Pseudo-nitzchia*, *Cylindrotheca*, and *Nitzchia*, varied significantly over time, but there was no significant effect of treatment.

**Fig. 6:**
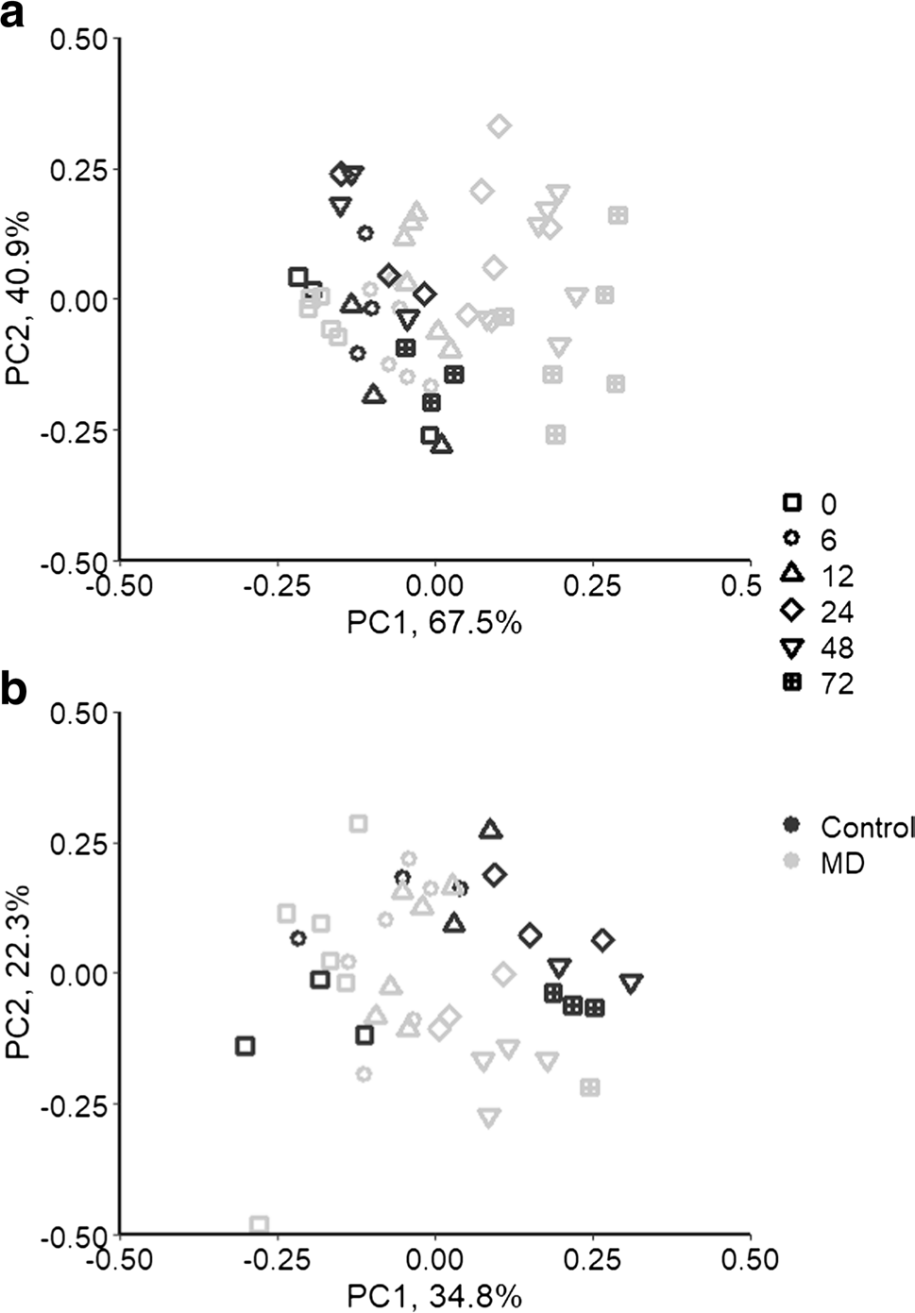
Robust PCA plots of the Aitchison distances for the prokaryote (**a**) and eukaryote (**b**) communities with the percent of the variation explained by each axis

**Fig. 7:**
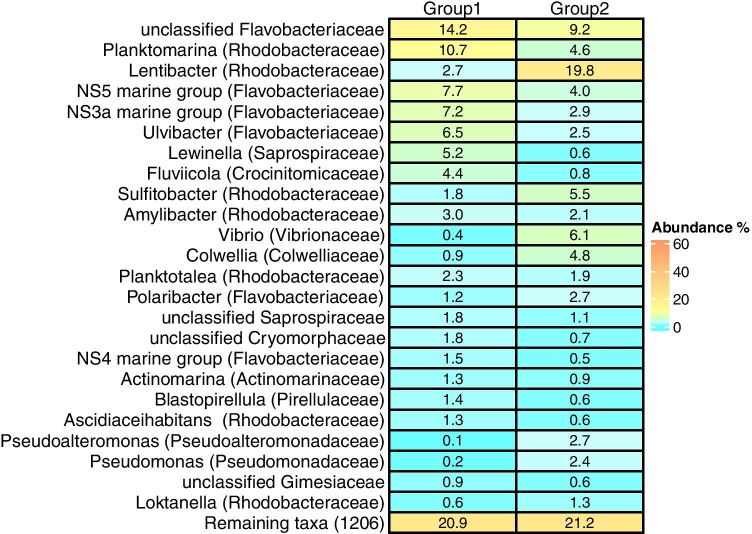
Heat map showing the most abundant genera, or families where lower taxonomic identification was not possible, in group 1 (all control samples and samples from MD enclosures from 0 h, 6 h, and 12 h) and group 2 (MD samples from 24 h, 48 h, and 72 h)

Poor amplification was observed for 18S rRNA genes, with a total of twelve samples excluded from the analysis, including five from 72 h. One sample was excluded from the 16S rRNA analysis. Robust PCA analysis of both genes showed different patterns of response between prokaryotic and eukaryotic communities (Fig. 6). Plots of eukaryote samples show the separation of samples by time along the PC1 axis, but minimal separation by treatment. This was supported by PERMANOVA analysis indicating a strong time effect (*p* < 0.001), but no treatment effect (*p* = 0.179) (Fig. 6b). PCA analysis of the prokaryotic community indicates little change in the control enclosures, but a strong change by 24 h in the presence of weathered MD (Fig. 6a). The PCA plot shows good separation along the PC1 axis, with all the control samples and samples from MD from 0 h, 6 h, and 12 h on the left (group 1), and the remaining samples (24–72 h) from MD enclosures on the right (group 2). A significant interaction between treatment and time was identified via PERMANOVA. ASVs were pooled at the genus level, and the most abundant genera (or families) were identified for the two groups of samples (Fig. 7). Both groups had high abundances of unclassified Flavobacteriacea. Group 1, representing the control samples and the early MD samples, also included high abundances of *Planktomarina* and the NS5 and NS3a marine groups from the Flavobacteriacea. In contrast, group 2, including only MD samples collected between 24 and 72 h, was dominated by *Lentibacter*, with *Vibrio*, *Sulfitobacter*, and *Colwellia* having much higher abundances than group 1.

## Discussion

Characterizing responses of entire marine microbial communities to partially weathered MD provides insights into what may happen when diesel enters a coastal ecosystem due to run-off. Weathering altered the chemical composition of the diesel, causing loss of volatile compounds before introduction to the enclosures. Although exposed to similar conditions, the three individual MD samples weathered slightly differently, representing a range of potential weathering which could occur under natural conditions. Despite the range of hydrocarbon concentrations in this experiment (maximum concentrations 0.5–4.4 µg mL^−1^), the responses of the microbial communities were relatively consistent and rapid, both in terms of abundance and diversity. This indicates that MD can alter the microbial community, even at low concentrations and after partial weathering.

Abundances of prokaryotes changed over time in both treatments. While increases in abundance in the control enclosures occurred immediately, peaking between 24 and 48 h, there was a lag of 24 h before abundances increased in the weathered MD enclosures. This timing corresponds to the shift in the microbial community from group 1 to group 2 samples. Studies have shown that once exposed to oil, bacterial communities can shift from a natural community to becoming dominated by oil-degrading bacteria [13–15], often with a lag, as the abundance of hydrocarbon-degrading microbes is low in uncontaminated communities.

*Planktomarina* and the NS5 and NS3a groups of Flavobacteriaceae are marine generalists found year-round in phytoplankton-rich marine communities [41–43]. They responded negatively to diesel, decreasing after 24 h, which concurs with previous oil exposure studies using crude oils [38, 44]. After 24 h, genera associated with oil exposure increased. Eight genera increased in group 2 samples compared to group 1, including *Lentibacter*, *Vibrio*, *Colwellia*, *Sulfitobacter*, *Pseudoalteromonas*, *Pseudomonas*, *Polaribacter*, and *Loktenella*. Several of these include species well known for oil degradation, while others may be opportunistic and take advantage of increased organic matter as phytoplankton abundances decrease. *Colwellia* has been observed to increase after oil spills, such as after *Deepwater Horizon* [15, 16], and in microcosm experiments using Arabian Light oil [45]. In addition, *Sulfitobacter* has been associated with early responses in oil spills [46]. In contrast, *Vibrio* is commonly associated with oil spills but is unlikely to degrade the oil itself [7]. The dominance of *Lentibacter*, which accounted for almost 20% of the MD community, may be related to increased dissolved organic nitrogen compounds as the abundances of phytoplankton decreased [47]. Increases in abundances and a shift in community structure after 24 h indicate a rapid response of the prokaryotic community to exposure to low concentrations of partially weathered MD. *Vibrio* and *Sulfitobacter* were quick to dominate the community when exposed to similar concentrations of unweathered diesel in a previous study [7]. This would indicate that these organisms are responding to the less volatile hydrocarbons remaining in the partially weathered diesel.

Decreases from phytoplankton may have fueled some increases in prokaryotic growth in later hours, although the drawdown of inorganic nutrients would suggest that the prokaryotes were growing on the carbon-rich diesel. In oil-exposed microbial communities, decreases in dinoflagellates, diatoms, and heterotrophic nanoflagellates have correlated with increases in the prokaryotic community [8]. Ortmann et al. [8] observed an increase in ciliates after 24–48 h, which graze on prokaryotes, whereas a slight increase in the abundance of the dinoflagellate grazer, *Gyrodinium*, was observed at 72 h in the current study. These organisms contribute significantly to metazooplankton diets [48]. The increase in *Gyrodinium* may also be attributed to their ability to ingest the oil, as *Gyrodinium spirale* has been found to ingest dispersed oil in surface waters during blooms [49].

Most phytoplankton and microzooplankton responded negatively to the addition of weathered MD relative to control communities (Figs. 4 and 5). In response to artificially weathered diesel, mesozooplankton and dinoflagellates decreased [12]. Nayar et al. [10] noted that higher concentrations of dissolved hydrocarbons from diesel (~ 1100 µg L^−1^) resulted in acute toxicity and decreased cell counts in autotrophs and phytoplankton, but at lower concentrations (~ 41 µg L^−1^), a stimulatory effect occurred. The peak concentrations in this study were closer to the higher concentrations in Nayar et al. [10] that resulted in inhibition of phytoplankton production and decreases in the diatoms *Skeletonema costatum* and *Thalassiosira*. Decreases in this study happened in both the control and weathered MD enclosures. However, by 72 h, there appeared to be recoveries solely in control enclosures, mostly due to increases of groups F and H. The 72 h time frame may have prevented observation of a larger recovery by the microeukaryotic community due to their slower growth rates compared to the prokaryotes.

Although there was poor sequencing success in the MD-exposed samples at later time points, there was no significant difference in community structure between partially weathered MD and control samples. The lack of significant differences in community structure between control and MD-exposed samples suggests that any toxic impact on the phytoplankton occurred equally across species. No stimulatory effect was observed in this study, possibly due to relatively high concentrations of hydrocarbons [10]. The mortality observed in this study may not occur under natural conditions where lack of boundaries may enable some of the larger plankton to actively avoid hydrocarbon-contaminated waters [50].

The addition of partially weathered MD to coastal waters containing indigenous microbial communities affected prokaryote and eukaryote communities differently. Control enclosures suggest a positive response to the establishment of enclosures for prokaryotes, while phytoplankton and microzooplankton decreased initially, followed by a recovery. Exposure to MD led to a rapid response in the prokaryote community, with increasing numbers corresponding to a shift in community structure being dominated by hydrocarbon-associated genera after 24 h. In contrast, eukaryotes did not recover in weathered MD enclosures, with no difference in community structure between treatments. These responses were observed over a range of hydrocarbon concentrations and without volatile hydrocarbons known to have toxic effects. Run-off events, where partially weathered diesel enters coastal waters, can occur after any rain event. This study demonstrates a strong, rapid response of the prokaryote community to hydrocarbons, suggesting biodegradation can reduce contaminated concentrations. Short-term, partially weathered diesel may be toxic to small phytoplankton and microzooplankton, but biodegradation and dilution through mixing may mitigate the impact.

## Supplementary Information

Below is the link to the electronic supplementary material.

Supplementary file1 (PDF 439 KB)

Supplementary file2 (XLSX 277 KB)

## Data Availability

The sequencing data have been deposited with links to BioProject accession number PRJNA678694 in the NCBI BioProject database, https://www.ncbi.nlm.nih.gov/sra/PRJNA678694.
