# Supplementary material for: Temperate Coastal Microbial Communities Rapidly Respond to Low Concentrations of Partially Weathered Diesel
Source: Microb Ecol. 2021 Dec 10;84(4):1122–32. doi: 10.1007/s00248-021-01939-w (PMC9747835; doi:10.1007/s00248-021-01939-w)
Supplement: Supplementary file 1 — Supplementary file1 (PDF 439 KB) [file 248_2021_1939_MOESM1_ESM.pdf]

Temperate coastal microbial communities rapidly respond to low concentrations of partially weathered diesel fuel

Microbial Ecology

Camilla M. Ryther[1], Gary Wohlgeschaffen[2], Brian J. Robinson[2] and Alice C. Ortmann[2]

[1]Dalhousie University (Biology Department), Halifax, NS, Canada Email: camilla.ryther@dal.ca

[2] Centre for Offshore Oil, Gas and Energy Research Laboratory, Bedford Institute of Oceanography, Fisheries and Oceans Canada, Dartmouth, NS, Canada Email: alice.ortmann@dfo-mpo.gc.ca

Supplemental Figures

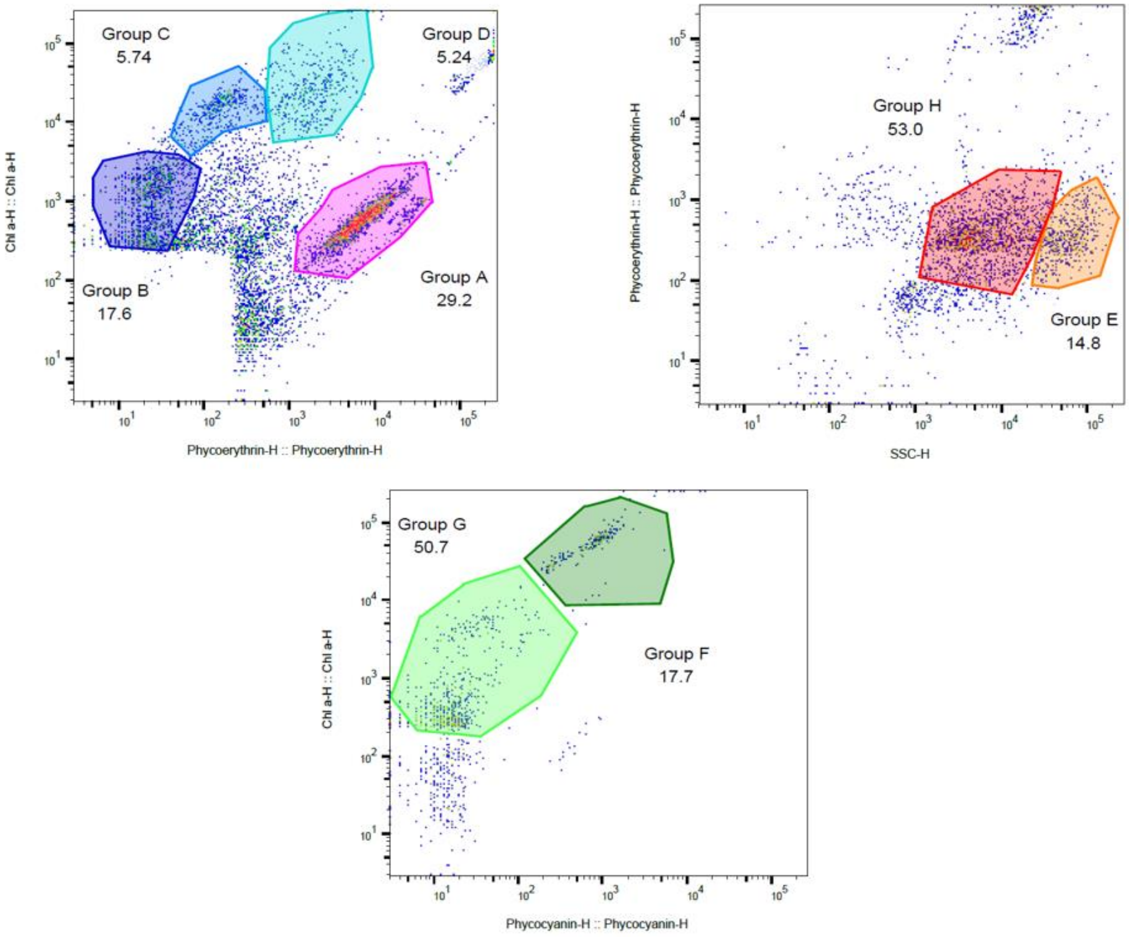

Fig. S1 Gates depicting 8 groups of small phytoplankton (<5 µm) based on fluorescence. Top left: Chl A vs. phycoerythrin Top right: Phycoerythrin vs. SSC-H Bottom: Chl A vs. phycocyanin. Sample shown is the low treatment from incubator A at t=0. Group A – phycoerythrin-containing cyanobacteria. Group B and C - likely different green algal flagellates. Group D may include diatoms. Group E and H may be cryptophytes. Group F and G possess phycocyanin containing cyanobacteria.

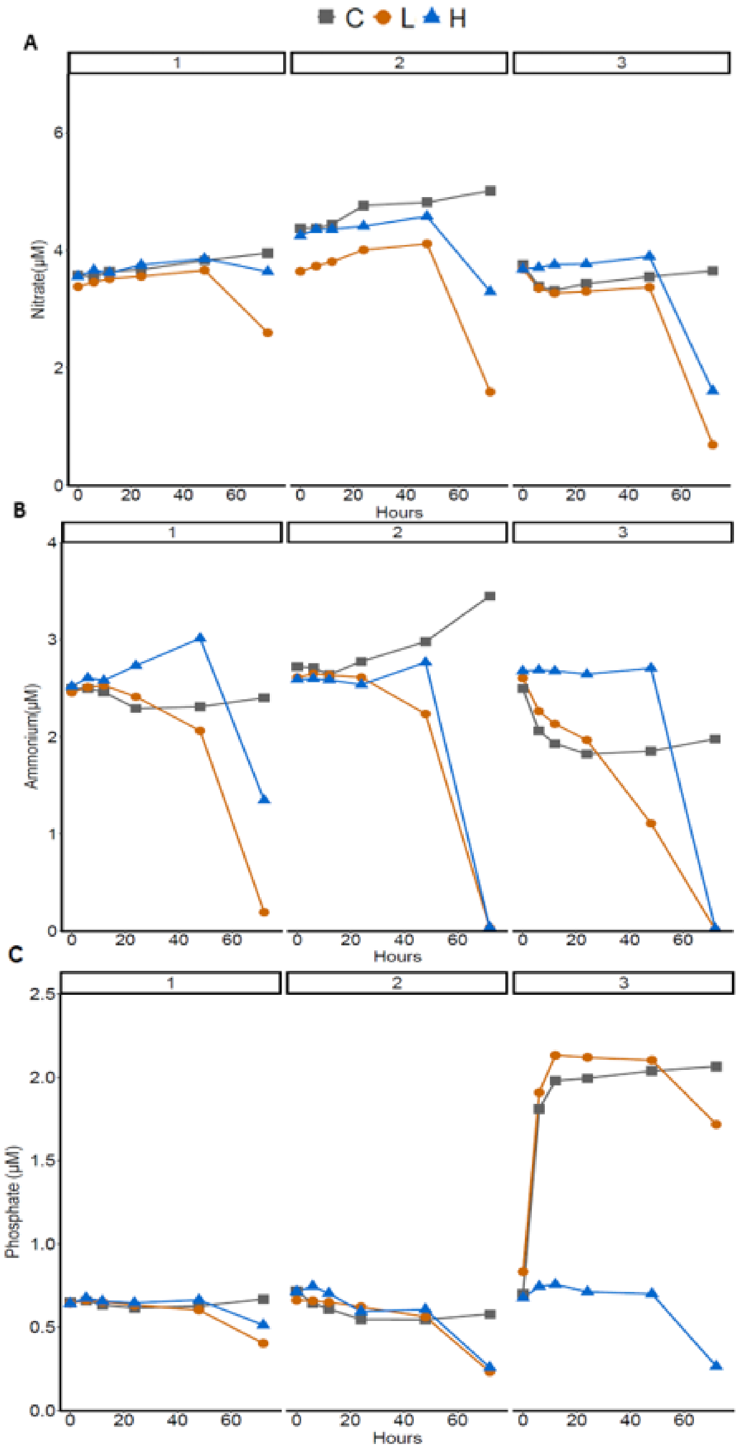

Fig. S2 Concentrations of Nitrate, Ammonium and Phosphate over time for each enclosure by incubator. Introduction of fresh water between 0 and 6 h in 2 enclosures resulted in the large shifts seen in the enclosures in Incubator 3.
